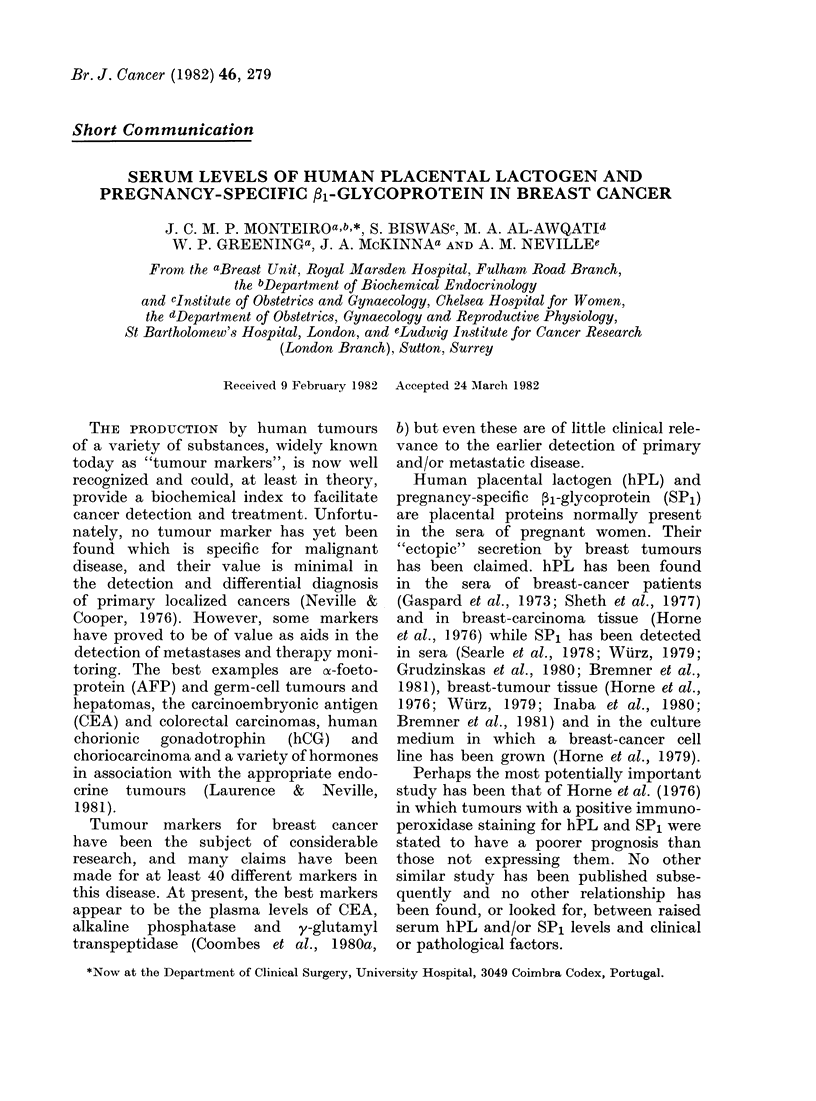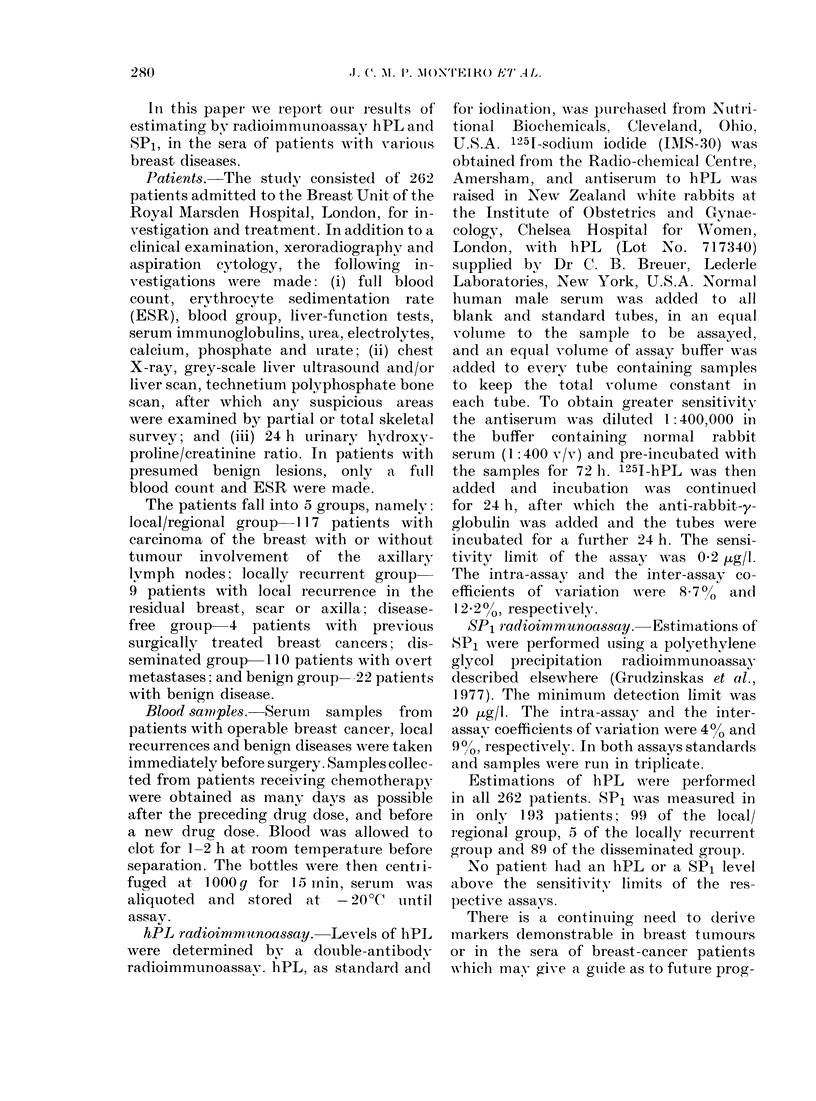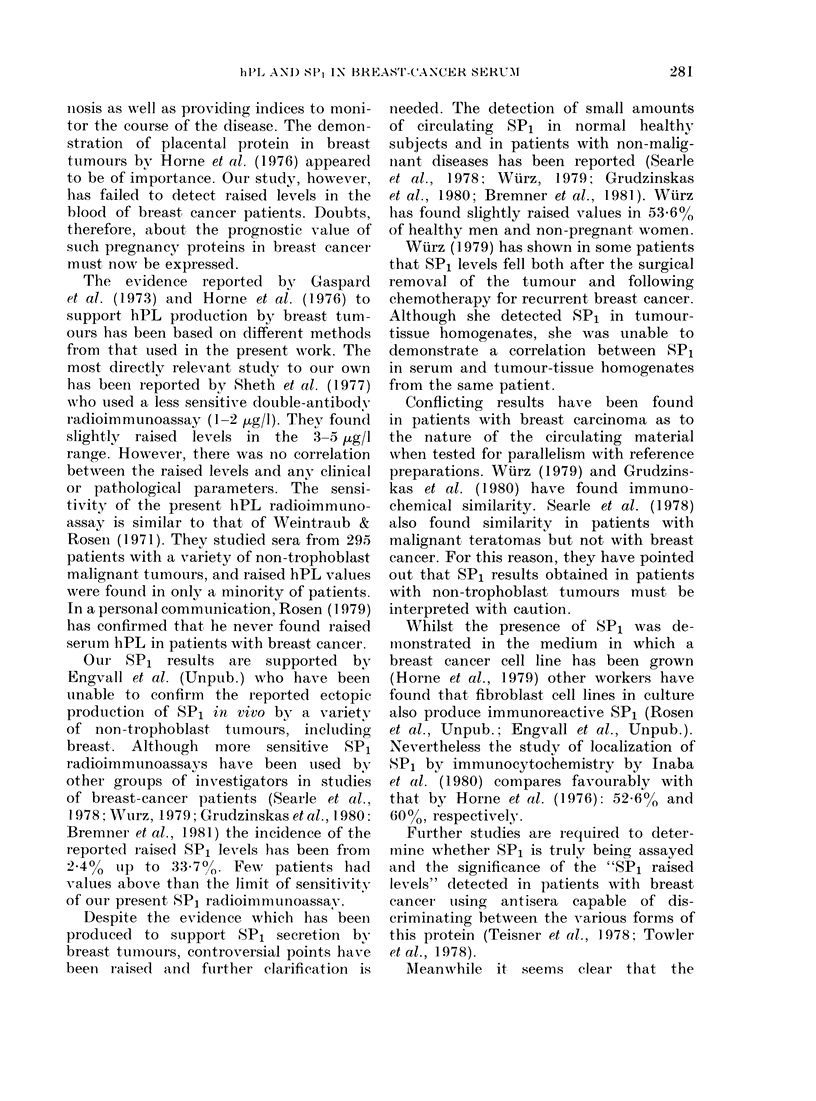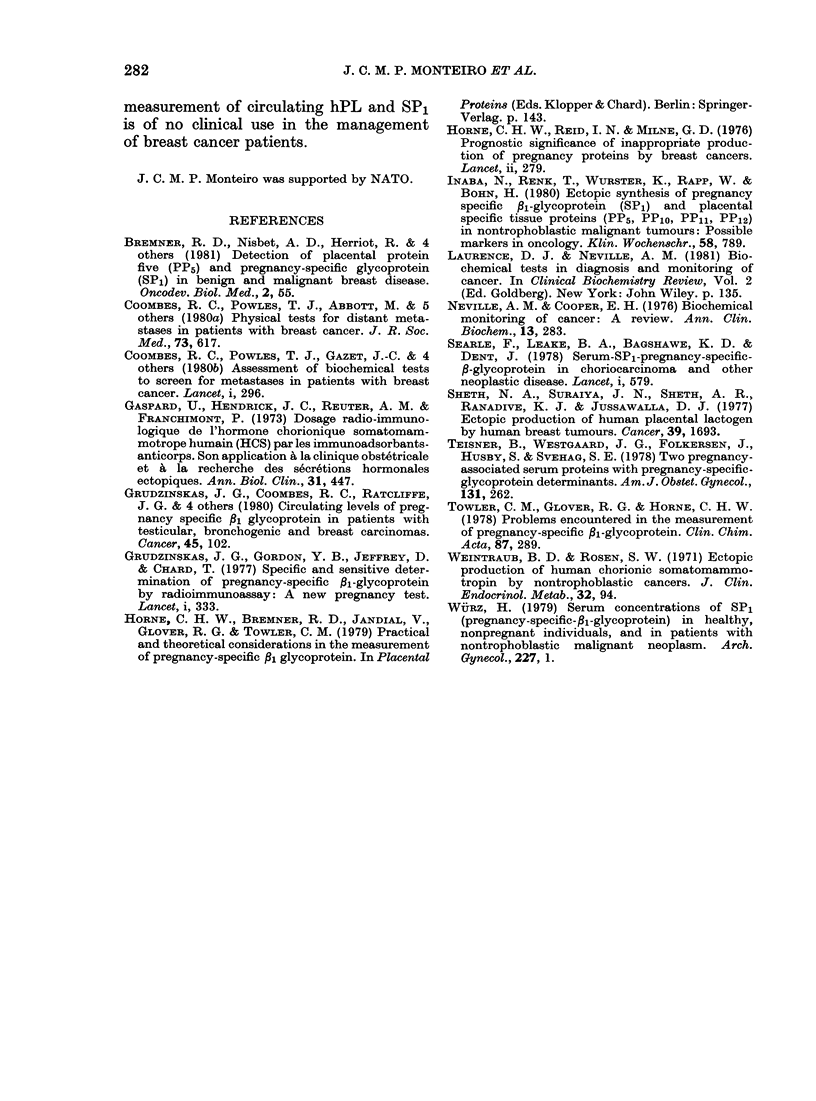# Serum levels of human placental lactogen and pregnancy-specific beta 1-glycoprotein in breast cancer.

**DOI:** 10.1038/bjc.1982.193

**Published:** 1982-08

**Authors:** J. C. Monteiro, S. Biswas, M. A. Al-Awqati, W. P. Greening, J. A. McKinna, A. M. Neville


*Br. J. Cancer* (1982) **46**, 279

***Short Communication***

### SERUM LEVELS OF HUMAN PLACENTAL LACTOGEN AND PREGNANCY-SPECIFIC $\beta_1$-GLYCOPROTEIN IN BREAST CANCER

J. C. M. P. MONTEIRO[a,b,*], S. BISWAS[c], M. A. AL-AWQATI[d]
W. P. GREENING[a], J. A. McKINNA[a] AND A. M. NEVILLE[e]

*From the [a]Breast Unit, Royal Marsden Hospital, Fulham Road Branch, the [b]Department of Biochemical Endocrinology and [c]Institute of Obstetrics and Gynaecology, Chelsea Hospital for Women, the [d]Department of Obstetrics, Gynaecology and Reproductive Physiology, St Bartholomew's Hospital, London, and [e]Ludwig Institute for Cancer Research (London Branch), Sutton, Surrey*

Received 9 February 1982 Accepted 24 March 1982

THE PRODUCTION by human tumours of a variety of substances, widely known today as "tumour markers", is now well recognized and could, at least in theory, provide a biochemical index to facilitate cancer detection and treatment. Unfortunately, no tumour marker has yet been found which is specific for malignant disease, and their value is minimal in the detection and differential diagnosis of primary localized cancers (Neville & Cooper, 1976). However, some markers have proved to be of value as aids in the detection of metastases and therapy monitoring. The best examples are α-foetoprotein (AFP) and germ-cell tumours and hepatomas, the carcinoembryonic antigen (CEA) and colorectal carcinomas, human chorionic gonadotrophin (hCG) and choriocarcinoma and a variety of hormones in association with the appropriate endocrine tumours (Laurence & Neville, 1981).

Tumour markers for breast cancer have been the subject of considerable research, and many claims have been made for at least 40 different markers in this disease. At present, the best markers appear to be the plasma levels of CEA, alkaline phosphatase and γ-glutamyl transpeptidase (Coombes *et al.*, 1980*a*, *b*) but even these are of little clinical relevance to the earlier detection of primary and/or metastatic disease.

Human placental lactogen (hPL) and pregnancy-specific $\beta_1$-glycoprotein ($SP_1$) are placental proteins normally present in the sera of pregnant women. Their "ectopic" secretion by breast tumours has been claimed. hPL has been found in the sera of breast-cancer patients (Gaspard *et al.*, 1973; Sheth *et al.*, 1977) and in breast-carcinoma tissue (Horne *et al.*, 1976) while $SP_1$ has been detected in sera (Searle *et al.*, 1978; Würz, 1979; Grudzinskas *et al.*, 1980; Bremner *et al.*, 1981), breast-tumour tissue (Horne *et al.*, 1976; Würz, 1979; Inaba *et al.*, 1980; Bremner *et al.*, 1981) and in the culture medium in which a breast-cancer cell line has been grown (Horne *et al.*, 1979).

Perhaps the most potentially important study has been that of Horne *et al.* (1976) in which tumours with a positive immunoperoxidase staining for hPL and $SP_1$ were stated to have a poorer prognosis than those not expressing them. No other similar study has been published subsequently and no other relationship has been found, or looked for, between raised serum hPL and/or $SP_1$ levels and clinical or pathological factors.

*Now at the Department of Clinical Surgery, University Hospital, 3049 Coimbra Codex, Portugal.

In this paper we report our results of estimating by radioimmunoassay hPL and $SP_1$, in the sera of patients with various breast diseases.

*Patients.*—The study consisted of 262 patients admitted to the Breast Unit of the Royal Marsden Hospital, London, for investigation and treatment. In addition to a clinical examination, xeroradiography and aspiration cytology, the following investigations were made: (i) full blood count, erythrocyte sedimentation rate (ESR), blood group, liver-function tests, serum immunoglobulins, urea, electrolytes, calcium, phosphate and urate; (ii) chest X-ray, grey-scale liver ultrasound and/or liver scan, technetium polyphosphate bone scan, after which any suspicious areas were examined by partial or total skeletal survey; and (iii) 24 h urinary hydroxyproline/creatinine ratio. In patients with presumed benign lesions, only a full blood count and ESR were made.

The patients fall into 5 groups, namely: local/regional group—117 patients with carcinoma of the breast with or without tumour involvement of the axillary lymph nodes; locally recurrent group—9 patients with local recurrence in the residual breast, scar or axilla; disease-free group—4 patients with previous surgically treated breast cancers; disseminated group—110 patients with overt metastases; and benign group—22 patients with benign disease.

*Blood samples.*—Serum samples from patients with operable breast cancer, local recurrences and benign diseases were taken immediately before surgery. Samples collected from patients receiving chemotherapy were obtained as many days as possible after the preceding drug dose, and before a new drug dose. Blood was allowed to clot for 1–2 h at room temperature before separation. The bottles were then centrifuged at 1000 *g* for 15 min, serum was aliquoted and stored at −20°C until assay.

*hPL radioimmunoassay.*—Levels of hPL were determined by a double-antibody radioimmunoassay. hPL, as standard and for iodination, was purchased from Nutritional Biochemicals, Cleveland, Ohio, U.S.A. $^{125}$I-sodium iodide (IMS-30) was obtained from the Radio-chemical Centre, Amersham, and antiserum to hPL was raised in New Zealand white rabbits at the Institute of Obstetrics and Gynaecology, Chelsea Hospital for Women, London, with hPL (Lot No. 717340) supplied by Dr C. B. Breuer, Lederle Laboratories, New York, U.S.A. Normal human male serum was added to all blank and standard tubes, in an equal volume to the sample to be assayed, and an equal volume of assay buffer was added to every tube containing samples to keep the total volume constant in each tube. To obtain greater sensitivity the antiserum was diluted 1:400,000 in the buffer containing normal rabbit serum (1:400 v/v) and pre-incubated with the samples for 72 h. $^{125}$I-hPL was then added and incubation was continued for 24 h, after which the anti-rabbit-$\gamma$-globulin was added and the tubes were incubated for a further 24 h. The sensitivity limit of the assay was 0·2 $\mu$g/l. The intra-assay and the inter-assay coefficients of variation were 8·7% and 12·2%, respectively.

*$SP_1$ radioimmunoassay.*—Estimations of $SP_1$ were performed using a polyethylene glycol precipitation radioimmunoassay described elsewhere (Grudzinskas *et al.*, 1977). The minimum detection limit was 20 $\mu$g/l. The intra-assay and the inter-assay coefficients of variation were 4% and 9%, respectively. In both assays standards and samples were run in triplicate.

Estimations of hPL were performed in all 262 patients. $SP_1$ was measured in in only 193 patients; 99 of the local/regional group, 5 of the locally recurrent group and 89 of the disseminated group.

No patient had an hPL or a $SP_1$ level above the sensitivity limits of the respective assays.

There is a continuing need to derive markers demonstrable in breast tumours or in the sera of breast-cancer patients which may give a guide as to future prognosis as well as providing indices to monitor the course of the disease. The demonstration of placental protein in breast tumours by Horne *et al.* (1976) appeared to be of importance. Our study, however, has failed to detect raised levels in the blood of breast cancer patients. Doubts, therefore, about the prognostic value of such pregnancy proteins in breast cancer must now be expressed.

The evidence reported by Gaspard *et al.* (1973) and Horne *et al.* (1976) to support hPL production by breast tumours has been based on different methods from that used in the present work. The most directly relevant study to our own has been reported by Sheth *et al.* (1977) who used a less sensitive double-antibody radioimmunoassay (1–2 $\mu$g/l). They found slightly raised levels in the 3–5 $\mu$g/l range. However, there was no correlation between the raised levels and any clinical or pathological parameters. The sensitivity of the present hPL radioimmunoassay is similar to that of Weintraub & Rosen (1971). They studied sera from 295 patients with a variety of non-trophoblast malignant tumours, and raised hPL values were found in only a minority of patients. In a personal communication, Rosen (1979) has confirmed that he never found raised serum hPL in patients with breast cancer.

Our $SP_1$ results are supported by Engvall *et al.* (Unpub.) who have been unable to confirm the reported ectopic production of $SP_1$ *in vivo* by a variety of non-trophoblast tumours, including breast. Although more sensitive $SP_1$ radioimmunoassays have been used by other groups of investigators in studies of breast-cancer patients (Searle *et al.*, 1978; Wurz, 1979; Grudzinskas *et al.*, 1980; Bremner *et al.*, 1981) the incidence of the reported raised $SP_1$ levels has been from 2·4% up to 33·7%. Few patients had values above than the limit of sensitivity of our present $SP_1$ radioimmunoassay.

Despite the evidence which has been produced to support $SP_1$ secretion by breast tumours, controversial points have been raised and further clarification is needed. The detection of small amounts of circulating $SP_1$ in normal healthy subjects and in patients with non-malignant diseases has been reported (Searle *et al.*, 1978; Würz, 1979; Grudzinskas *et al.*, 1980; Bremner *et al.*, 1981). Würz has found slightly raised values in 53·6% of healthy men and non-pregnant women.

Würz (1979) has shown in some patients that $SP_1$ levels fell both after the surgical removal of the tumour and following chemotherapy for recurrent breast cancer. Although she detected $SP_1$ in tumour-tissue homogenates, she was unable to demonstrate a correlation between $SP_1$ in serum and tumour-tissue homogenates from the same patient.

Conflicting results have been found in patients with breast carcinoma as to the nature of the circulating material when tested for parallelism with reference preparations. Würz (1979) and Grudzinskas *et al.* (1980) have found immunochemical similarity. Searle *et al.* (1978) also found similarity in patients with malignant teratomas but not with breast cancer. For this reason, they have pointed out that $SP_1$ results obtained in patients with non-trophoblast tumours must be interpreted with caution.

Whilst the presence of $SP_1$ was demonstrated in the medium in which a breast cancer cell line has been grown (Horne *et al.*, 1979) other workers have found that fibroblast cell lines in culture also produce immunoreactive $SP_1$ (Rosen *et al.*, Unpub.; Engvall *et al.*, Unpub.). Nevertheless the study of localization of $SP_1$ by immunocytochemistry by Inaba *et al.* (1980) compares favourably with that by Horne *et al.* (1976): 52·6% and 60%, respectively.

Further studies are required to determine whether $SP_1$ is truly being assayed and the significance of the "$SP_1$ raised levels" detected in patients with breast cancer using antisera capable of discriminating between the various forms of this protein (Teisner *et al.*, 1978; Towler *et al.*, 1978).

Meanwhile it seems clear that the

**measurement of circulating hPL and $SP_1$ is of no clinical use in the management of breast cancer patients.**

J. C. M. P. Monteiro was supported by NATO.

### REFERENCES

BREMNER, R. D., Nisbet, A. D., Herriot, R. & 4 others (1981) Detection of placental protein five ($PP_5$) and pregnancy-specific glycoprotein ($SP_1$) in benign and malignant breast disease. *Oncodev. Biol. Med.*, **2**, 55.

COOMBES, R. C., POWLES, T. J., ABBOTT, M. & 5 others (1980*a*) Physical tests for distant metastases in patients with breast cancer. *J. R. Soc. Med.*, **73**, 617.

COOMBES, R. C., POWLES, T. J., GAZET, J.-C. & 4 others (1980*b*) Assessment of biochemical tests to screen for metastases in patients with breast cancer. *Lancet*, i, 296.

GASPARD, U., HENDRICK, J. C., REUTER, A. M. & FRANCHIMONT, P. (1973) Dosage radio-immunologique de l'hormone chorionique somatomammotrope humain (HCS) par les immunoadsorbants-anticorps. Son application à la clinique obstétricale et à la recherche des sécrétions hormonales ectopiques. *Ann. Biol. Clin.*, **31**, 447.

GRUDZINSKAS, J. G., COOMBES, R. C., RATCLIFFE, J. G. & 4 others (1980) Circulating levels of pregnancy specific $\beta_1$ glycoprotein in patients with testicular, bronchogenic and breast carcinomas. *Cancer*, **45**, 102.

GRUDZINSKAS, J. G., GORDON, Y. B., JEFFREY, D. & CHARD, T. (1977) Specific and sensitive determination of pregnancy-specific $\beta_1$-glycoprotein by radioimmunoassay: A new pregnancy test. *Lancet*, i, 333.

HORNE, C. H. W., BREMNER, R. D., JANDIAL, V., GLOVER, R. G. & TOWLER, C. M. (1979) Practical and theoretical considerations in the measurement of pregnancy-specific $\beta_1$ glycoprotein. In *Placental Proteins* (Eds. Klopper & Chard). Berlin: Springer-Verlag. p. 143.

HORNE, C. H. W., REID, I. N. & MILNE, G. D. (1976) Prognostic significance of inappropriate production of pregnancy proteins by breast cancers. *Lancet*, ii, 279.

INABA, N., RENK, T., WURSTER, K., RAPP, W. & BOHN, H. (1980) Ectopic synthesis of pregnancy specific $\beta_1$-glycoprotein ($SP_1$) and placental specific tissue proteins ($PP_5$, $PP_{10}$, $PP_{11}$, $PP_{12}$) in nontrophoblastic malignant tumours: Possible markers in oncology. *Klin. Wochenschr.*, **58**, 789.

LAURENCE, D. J. & NEVILLE, A. M. (1981) Biochemical tests in diagnosis and monitoring of cancer. In *Clinical Biochemistry Review*, Vol. 2 (Ed. Goldberg). New York: John Wiley. p. 135.

NEVILLE, A. M. & COOPER, E. H. (1976) Biochemical monitoring of cancer: A review. *Ann. Clin. Biochem.*, **13**, 283.

SEARLE, F., LEAKE, B. A., BAGSHAWE, K. D. & DENT, J. (1978) Serum-$SP_1$-pregnancy-specific-$\beta$-glycoprotein in choriocarcinoma and other neoplastic disease. *Lancet*, i, 579.

SHETH, N. A., SURAIYA, J. N., SHETH, A. R., RANADIVE, K. J. & JUSSAWALLA, D. J. (1977) Ectopic production of human placental lactogen by human breast tumours. *Cancer*, **39**, 1693.

TEISNER, B., WESTGAARD, J. G., FOLKERSEN, J., HUSBY, S. & SVEHAG, S. E. (1978) Two pregnancy-associated serum proteins with pregnancy-specific-glycoprotein determinants. *Am. J. Obstet. Gynecol.*, **131**, 262.

TOWLER, C. M., GLOVER, R. G. & HORNE, C. H. W. (1978) Problems encountered in the measurement of pregnancy-specific $\beta_1$-glycoprotein. *Clin. Chim. Acta*, **87**, 289.

WEINTRAUB, B. D. & ROSEN, S. W. (1971) Ectopic production of human chorionic somatomammotropin by nontrophoblastic cancers. *J. Clin. Endocrinol. Metab.*, **32**, 94.

WÜRZ, H. (1979) Serum concentrations of $SP_1$ (pregnancy-specific-$\beta_1$-glycoprotein) in healthy, nonpregnant individuals, and in patients with nontrophoblastic malignant neoplasm. *Arch. Gynecol.*, **227**, 1.